# Supplementary material for: Exploring Potential Mechanisms of Fludioxonil Resistance in Fusarium oxysporum f. sp. melonis
Source: J Fungi (Basel). 2022 Aug 11;8(8):839. doi: 10.3390/jof8080839 (PMC9409840; doi:10.3390/jof8080839)
Supplement: Supplementary file 1 [file jof-08-00839-s001.zip › jof-1766206-SM.pdf]

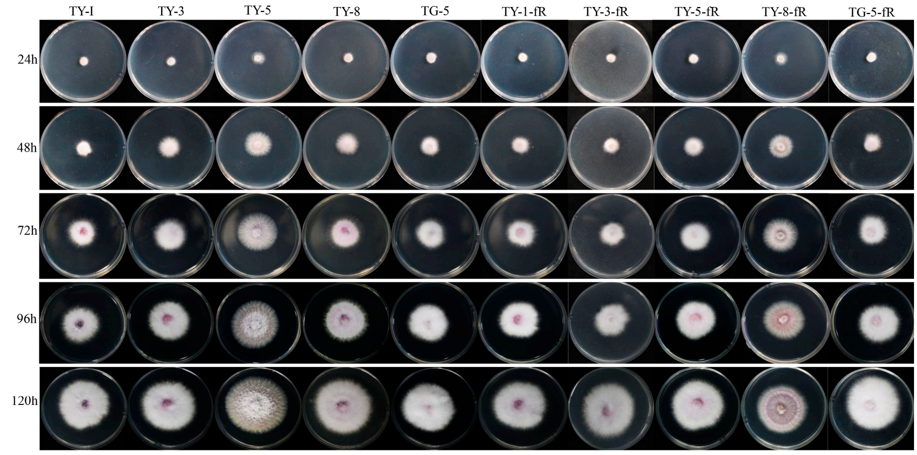

Figure S1. Mycelial growth of five fludioxonil-resistant mutants of FOM and their parental isolates growing on PDA at 24, 48, 72, 96, and 120 h post-inoculation (hpi).

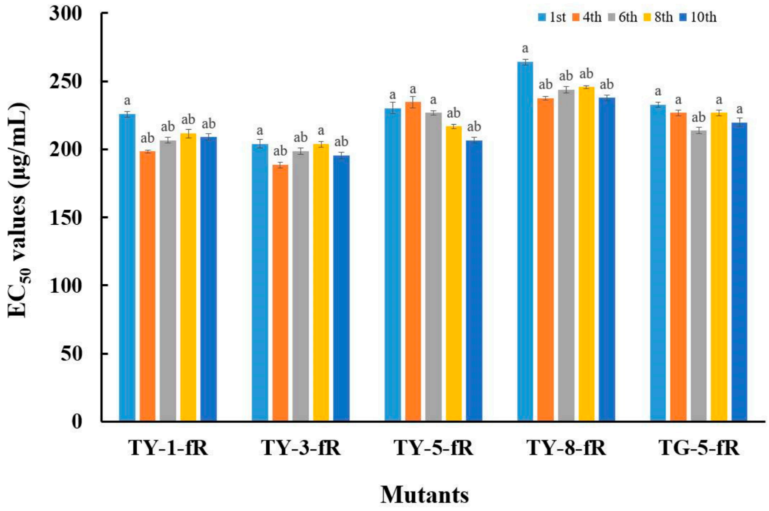

Figure S2. Heritable stability of five fludioxonil-resistant mutants of FOM assessed according to the variation in their fludioxonil EC_{50} values. Data are the means of five replicates ± SE. Error bars indicate one standard error of the mean (SE) as calculated from the mean of two separate experiments, while different letters above columns indicate significant differences according to Fisher's least significant difference test ($p \leq 0.05$).

Table S1. Mutations in the MAP kinase protein sequence of fludioxonil-resistant mutants of *F. graminearum*, *F. asiaticum*, and FOM were reported in China.

Species.	Resource.	Resistance type*	Genes.	Mutations.	Reference..
F. graminearum.	Laboratory.	HR.	*FgOs1.*	K215T, K411R.	[18]
F. asiaticum.	Field.	HR.	*FgOs1.*	R753 stop.	[34]
F. asiaticum.	Field.	HR.	*FgOs1.*	Q927 stop.	[34]
F. asiaticum.	Field.	MR / HR.	*FgOs1.*	No mutation.	[34]
F. asiaticum.	Laboratory.	HR.	*FgOs1.*	P1109S.	[34]
F. asiaticum.	Laboratory.	HR.	*FgOs1.*	P1161H.	[34]
F. asiaticum.	Laboratory.	HR.	*FgOs4.*	C928Y.	[34]

F. graminearum.	Laboratory.	HR.	*FgOs5.*	K192R.	[18]..
F. graminearum.	Laboratory.	HR.	*FgOs5.*	520 stop.	[18]..
F. graminearum.	Laboratory.	HR.	*FgOs5.*	K293R.	[18]..
F. graminearum.	Laboratory.	HR.	*FgOs5.*	K411R.	[18]..
F. asiaticum.	Laboratory.	HR.	*FgOs5.*	S39T.	[34]..
F. asiaticum.	Laboratory.	HR.	*FgOs5.*	S90T.	[34]..
F. asiaticum.	Laboratory.	HR.	*FgOs5.*	S116T.	[34]..
F. asiaticum.	Laboratory.	HR.	*FgOs5.*	L350P.	[34]..
F. asiaticum.	Laboratory.	HR.	*FgOs5.*	R598T.	[34]..
F. asiaticum.	Laboratory.	HR.	*FgOs5.*	R602G.	[34]..
F. oxysporum.	Laboratory.	HR.	*FoOs1.*	S564P, E702Q.	Current study..
F. oxysporum.	Laboratory.	HR.	*FoOs1.*	E702Q, A896T.	Current study..
F. oxysporum.	Laboratory.	HR.	*FoOs1.*	E702Q, A896T.	Current study..
F. oxysporum.	Laboratory.	HR.	*FoOs1.*	R66A, N537T, and E702Q.	Current study..
F. oxysporum.	Laboratory.	HR.	*FoOs1.*	N537T and E702Q.	Current study..

* Resistance level indicating degree of sensitivity to fludioxonil: S = sensitive, LR = low resistance, MR = medium resistance, and HR = high resistance.
